# Buspirone Nanovesicular Nasal System for Non-Hormonal Hot Flushes Treatment

**DOI:** 10.3390/pharmaceutics10030082

**Published:** 2018-07-03

**Authors:** Elka Touitou, Hiba Natsheh, Shaher Duchi

**Affiliations:** The Institute for Drug Research, School of Pharmacy, Faculty of Medicine, The Hebrew University of Jerusalem, P.O. Box 12065, Jerusalem 91120, Israel; hiba.natsheh@mail.huji.ac.il (H.N.); shaherd75@gmail.com (S.D.)

**Keywords:** nanovesicular nasal carrier, nasal delivery system, buspirone, hot flushes, ovariectomized rat

## Abstract

The aim of this work was to design and characterize a new nanovesicular nasal delivery system (NDS) containing buspirone, and investigate its efficiency in an animal model for the treatment of hot flushes. The presence of multilamellar vesicles with a mean size distribution of 370 nm was evidenced by transition electron microscopy (TEM), cryo-scanning electron microscopy (Cryo-SEM), and dynamic light scattering (DLS) tests. Pharmacodynamic evaluation of the nasal treatment efficacy with the new system was carried out in ovariectomized (OVX) rat—an animal model for hot flushes—and compared with other treatments. We found that the nasal administration of a buspirone NDS resulted in a significant reduction in tail skin temperature (TST). This effect was not observed in the control buspirone-treated groups. Buspirone levels in the plasma and brain of nasally-treated normal rats were quantified and compared with those of rats that had received oral administration by a LC-MS/MS assay. A significantly higher bioavailability was achieved with the new treatment relative to an oral administration of the same drug dose. No pathological changes in the nasal cavity were observed following sub-chronic nasal administration of buspirone NDS. In conclusion, the data of our investigation show that buspirone in the new nanovesicular nasal carrier could be considered for further studies for the development of a treatment for the hot flushes ailment.

## 1. Introduction

Currently, most of therapeutic products for hot flushes are based on hormone therapy (HT), involving the administration of estrogen alone or in combination with progesterone. There is a growing demand for a safer treatment alternative to HT for hot flushes [1].

Drugs affecting serotonin levels were investigated for non-hormonal therapies for hot flushes. In a previous study, we reported that buspirone administrated in a transdermal system has efficiently treated hot flushes in an animal model [2]. Buspirone—a 5-HT1A agonist—is currently administered orally and indicated for the treatment of generalized anxiety disorder (GAD). Buspirone HCl belongs to biopharmaceutics classification system (BCS) class I, being highly soluble and highly permeable. However, oral administration of this drug is associated with low bioavailability: ~4% due to an extensive first-pass metabolism [3]. In addition, this centrally acting drug lacks the ability to penetrate the blood–brain barrier (BBB).

Nasal administration, being able to circumvent the first pass metabolism and bypass the BBB, could be a promising alternative for the oral administration of buspirone. Despite this, the nasal delivery of many molecules is poor, due to the low permeability of the nasal mucosa [4,5].

Touitou and Illum emphasized the role of the carrier in the design of an efficient nasal product [4]. To overcome the above drawbacks, we propose here the nasal administration of buspirone incorporated in a new nanovesicular delivery system (NDS) to be tested in a hot flushes animal model. We have shown that the new vesicular nasal carrier enhanced the pharmacodynamic effect of a number of other drugs [6,7,8].

Various aspects of nasal delivery of buspirone were previously studied [9,10,11,12]. Khan et al. investigated the nasal clearance, bioavailability, and delivery to brain of buspirone in chitosan mucoadhesive nasal formulation in vivo [9,10,11]. Mathure et al. studied the ex vivo permeation of buspirone niosomes gel through sheep nasal mucosa [12].

In this work, we designed and characterized buspirone NDS, tested its pharmacodynamic effect in an ovariectomized (OVX) animal model for hot flushes, and measured the drug levels in brain and plasma. The safety of the local application of the nanovesicular system on the animal nasal cavity was also examined.

To our knowledge, this pharmacodynamic effect of nasally administrated buspirone in a nanovesicular carrier has not been previously investigated.

## 2. Materials and Methods

### 2.1. Materials

Buspirone HCl was a gift from Unipharm, Israel. Ethinylestradiol (EE) was purchased from Sigma (Jerusalem, Israel). Phosphatidylcholine phospholipid, Phospholipon 90 G, was bought from Lipoid GmbH (Berlin, Germany). Propylene glycol and Vitamin E (Tocopheryl acetate) were acquired from Tamar (Rishon Lezion, Israel). Ethanol absolute (Gadot, Netanya, Israel) was acquired from the Hebrew University warehouse. All of the other materials used in this work were of analytical or pharmaceutical grade.

### 2.2. Animals

All of the procedures performed on animals were conducted according to The National Institutes of Health regulations and approved by the Committee for Animal Care and Experimental Use of the Hebrew University of Jerusalem, Ethics No. MD-11-12833-3 (2011–2015).

Sprague–Dawley female rats (weighing 250–360 g) were purchased from Harlan (Rehovot, Israel). The rats were housed in separate cages and were maintained on a 12 h light, 12 h dark cycle, with lights on from 07:00 to 19:00 daily with free access to food and water.

The administration of buspirone from all of the systems was carried out under short anesthesia with isoflurane, including the animals in the control groups. This was sufficient to keep the rats sedated for a short period of 1–2 min during the instillation of nasal formulations to prevent sneezing.

### 2.3. Preparation and Characterization of Buspirone NDS

The new carrier was composed of phospholipid: propylene glycol: ethanol at the ratio 1:4:3 per weight. Additional components were vitamin E and water [6].

Buspirone NDS was prepared by a simple mixing method using an overhead Heidolph^®^ stirrer (Heidolph Digital 200 RZR-2000, Schwabach, Germany). Briefly, phospholipid was dissolved in ethanol; then, propylene glycol and vitamin E were added. Buspirone HCl aqueous solution was then added slowly with continuous mixing; the nanovesicles were generated at this stage.

In this work, we present the results obtained with 3% *w*/*w* buspirone NDS. The system was tested for drug chemical content, the presence of nanovesicles, the size distribution of vesicles, viscosity, pH, and three months’ stability.

#### 2.3.1. Drug Chemical Content

The concentration of buspirone in the system was quantified by HPLC (Merck-Hitachi D-7000 equipped with an L-7400 variable UV detector, L-7300 column oven, L-7200 auto-sampler, L-7100 pump, and an Hardware Security Module (HSM) computerized analysis program, Tokyo, Japan). The drug concertation in the samples was determined using a modified method described by Foroutan et al. [13]. The chromatographic conditions were set as follows: UV detection 240 nm, a Nucleosil C18 125 mm × 4 mm 5 micron column with a mobile phase of acetonitrile: phosphate buffer 0.01 M pH 3.5 (40:60, *v*/*v*) at a flow of 1 mL/min.

#### 2.3.2. Visualization of Vesicles by Transition Electron Microscopy (TEM) and Cryo-Scanning Electron Microscopy (Cryo-SEM)

For transition electron microscopy (TEM) visualization, one day before the examination, the system was diluted 1:10 with suitable diluent and stained with 1% aqueous solution of phosphotungstic acid (PTA), dried at room temperature for 20 min, and viewed under the microscope (Philips TECHNAI CM 120 electron microscope, Eindhoven, The Netherlands) at 26.5–110 k-fold enlargement.

For cryo-scanning electron microscopy (cryo-SEM) visualization, specimen preparation was performed with a BAF-060 system (BalTec AG, Balzers, Liechtenstein). A small drop of the buspirone NDS was placed on an electron microscopy copper grid and sandwiched between two gold planchettes. The “sandwich” was plunged into liquid ethane at its freezing point, transferred into liquid nitrogen, and inserted into a sample fracture block that had been pre-cooled by liquid nitrogen. The block was split open to fracture the frozen sample drop. A Pt–C conductive thin film of 4 nm was deposited on the surfaces (at a 90° angle). The coated specimens were transferred under vacuum by a BalTec VCT100 shuttle that had been pre-cooled with liquid nitrogen into a Zeiss Ultra Plus High-Resolution Scanning Electron Microscope (HR-SEM) (Oberkochen, Germany), and maintained at −150 °C. The microscopic examination was performed under 3000-fold enlargement.

#### 2.3.3. Vesicles Size Distribution by Dynamic Light Scattering (DLS)

Buspirone NDS was analyzed using a Malvern Zetasizer-nano, ZEN 3600, Malvern Instruments, Malvern, UK. The system was diluted 1:500 with suitable diluent one hour prior to measurement. Three batches of each system were tested. Each batch was analyzed by intensity, three times, at 25 °C. The duration and the set position of each measurement were fixed automatically by the apparatus.

#### 2.3.4. Viscosity and pH of the System

The viscosity of the nanovesicular system was measured by Brookfield DV III Rheometer -LV (Brookfield engineering labs, Stoughton, MA, USA), spindle 18 and a small sample adaptor, at a rotation speed of 30 rpm.

The pH measurements were performed by a Fisher pH meter (Fisher Instruments, Pittsburgh, PA, USA). The system was diluted with double distilled water 1:5. All of the measurements were duplicated.

#### 2.3.5. Stability Test for Buspirone NDS

Changes in drug chemical content, structure, and size distribution of the nanovesicles, viscosity, and pH of the system were measured and compared to zero time values following three months storage at room temperature (RT).

### 2.4. Pharmacodynamic Effect Evaluation in Animal Model

#### 2.4.1. Animal Model

The protocol for the animal model and the experiments was conducted according to The National Institutes of Health regulations and approved by the Committee for Animal Care and Experimental Use of the Hebrew University of Jerusalem, as above in Section 2.2.

The effect of nasal administration of buspirone NDS on a hot flushes animal model was tested in bilateral ovariectomized rats (OVX). This is a model for estrogen deficiency-associated thermoregulatory dysfunction. The treatment effect was evaluated by monitoring the changes in tail skin temperature (TST) [2].

A total of 71 rats weighing 250–330 g underwent bilateral ovariectomies (OVX) (*n* = 63) or sham surgeries (*n* = 8) (which left their ovaries intact) at Harlan Biotech (Rehovot, Israel) followed by a recovery period of two weeks. No surgical or medical complications were observed.

Sixteen rats were used to test the animal model (including the eight sham and eight OVX), and 55 OVX rats were used for testing various treatments.

To test the reliability of the OVX animal model, the increase in the TST in OVX rats was assessed as compared with sham animals. The TST values for sham animals were considered the normal values [14].

#### 2.4.2. Treatments and TST Measurements

For testing various treatments, 48 OVX animals were divided into six groups (*n* = 8/group) as follows: single administration of 3 mg/kg buspirone from NDS as compared to nasal aqueous solution (NAQ), oral aqueous solution (PO), and subcutaneous injection (SC). In addition, the effect of buspirone NDS was evaluated compared to the positive control, ethinylestradiol (EE), which was administrated subcutaneously to OVX rats at a dose of 0.3 mg/kg once daily for seven days. Untreated OVX rats served as control. A 3% *w*/*w* buspirone aqueous solution was used as the nasal or oral control system, while a 0.3% *w*/*w* buspirone solution in normal saline was used for SC administration. The 0.03% *w*/*w* ethinylestradiol SC solution was prepared in sesame oil. The administrated volumes were calculated to achieve a dose of 3 mg/kg buspirone and 0.3 mg/kg EE to each animal.

The experiments were carried out in two replicates; each replicate included four rats for each treatment group. The intra and interobserver variations were ≤4.6% and 3.9%, respectively. The drug dose was chosen following the evaluation of the dose- effect relationship (data is not shown).

TST was measured using Thermalert TH-5 (Physitemp Instruments Inc., Clifton, NJ, USA). A thermocouple skin sensor probe SST-1 (Physitemp Instruments Inc., Clifton, NJ, USA) was fixed on the dorsal surface of the tail approximately one cm away from the base, and the animals were retained in a flat-bottomed restraint during the 30–60 s sampling period. All of the measurements were performed from 10:00 to 15:00 and at 21.5 ± 0.1 °C.

On the day of the experiment, the rats were acclimatized in the experiment room for two hours. TST values were recorded before treatment (baseline) and 30 min, 60 min, 120 min, 180 min, and 240 min after treatment.

The following parameters were used to evaluate the effect of the various treatments: the average TST value (TSTave, °C) for each time point was the average of the readings recorded in the two experimental replicates for animals in the same treatment group [15]. The ∆TST value for each treatment at a certain time point was obtained by subtracting TSTave at baseline from the value at that time point. The duration of effect is the time period (min) in which TSTave is statistically different from the TST at baseline [16].

As a next step, we determined the onset of the action of buspirone NDS by measuring the TST values of OVX rats each minute following the treatment (*n* = 7).

### 2.5. Determination of Buspirone Levels in Rat Plasma and Brain

The concentration of buspirone in the plasma and brain tissue of normal animals at various time points was measured post-dose of 3 mg/kg drug nasal administration in NDS and oral administration (PO).

#### 2.5.1. Drug Concentration in Plasma Measurement

Ten rats were randomly divided equally into two groups of five animals. Blood samples of 400–500 µL were collected from tail 5 min before treatment (zero-time point) and 5 min, 10 min, 20 min, 30 min, 60 min, 120 min, and 240 min after drug administration.

Briefly, blood samples were centrifuged at 3000 rpm for 10 min at room temperature, and plasma was transferred and stored at −20 °C until assayed. On the day of analysis, the samples were thawed, and buspirone was extracted from plasma by a modified protein precipitation method described by Foroutan et al. [13]. One hundred microliters of plasma samples were extracted with 125 µL acetonitrile and diluted with 275 µL of water. Samples were centrifuged at 14,000 rpm for 5 min at room temperature. Supernatants were filtered and injected into LC-MS/MS (Thermo Scientific, San Jose, CA, USA). 

#### 2.5.2. Drug Concentration in Brain Tissue Measurement

Sixteen rats were randomly divided into four equal groups for testing two time points and two treatments. Animals were sacrificed 10 min or 30 min after administration. Brains were collected, immediately weighed, and kept at −70 °C until analysis. Brain tissues were purified by a modified liquid–liquid extraction method described by Lai et al. [17]. On the day of analysis, the brain tissues were thawed and homogenized with 2 mL of water/g brain tissue. The homogenates were alkalinized with 10% *w*/*v* NaOH solution. Buspirone was extracted with 5 mL a mixture (4:1) of hexane and ethyl acetate. Samples were then centrifuged at 4000 rpm for 45 min at 4 °C. Supernatants were collected and evaporated at room temperature. Dried residues were reconstituted with mobile phase, and centrifuged at 14,000 rpm for 10 min at room temperature. Final supernatants were filtered and injected into LC-MS/MS. 

#### 2.5.3. Buspirone LC-MS/MS Assay

Buspirone content in plasma and brain was quantified by a specific validated LC-MS/MS method according to the FDA regulation guidelines of bioanalytical validation. A Kinetex™ column (2.6 µm Minibore C18 50 × 2.1 mm, Phenomenex^®,^ Torrance, CA, USA) was used. Flow rate and injection volume were 400 µL/min and 5 µL, respectively. At these defined conditions, the retention time of buspirone was 1.33 min. Buspirone plasma levels were expressed in ng/mL, and in ng/g in brain tissue. Standard calibration curves of buspirone hydrochloride were prepared with plasma and brain homogenates spiked with known amounts of drug (1–1000 ng/mL and 100–500 ng/g, respectively). For the standard calibration curve, each concentration was injected five times, and the experiment was duplicated. The inter-coefficients and intra-coefficients of variation were <2.2% and 4.0%, respectively. The sensitivity of the method was 1 ng/mL, and the recovery was 78% and 96.3% for plasma and brain, respectively.

The following parameters were used to evaluate the concentration profile in plasma: C_max_, T_max_, and AUC0-240 (from zero to 240 min). The AUC0−240 _[NDS]_ and AUC0-240 _[PO]_ represent the means of individual AUC0-240 from nasal and oral experimental groups, respectively. The area under the curve of plasma concentration was calculated using the linear trapezoidal rule. All of the pharmacokinetic parameters were calculated using a windows-based program for noncompartmental analysis of pharmacokinetic data, NCOMP; version 3.1 11-SEP-97 in (c) 1996-7 Fox Chase Cancer Center (Philadelphia, PA, USA).

The relative bioavailability (F %) was calculated according to the following equation:

F % = [(AUC0 − 240 _[NDS]_ × DOSE_PO_)]/[(AUC0 − 240 _[PO]_ × DOSE _NDS_)] × 100

### 2.6. Local Safety Assessment

In this experiment, we evaluated the effect of buspirone NDS on the nasal cavity in rats by a method previously described by Duchi et al. [7,8]. In brief, six rats were divided into three equal groups. Rats in the nasal administration groups received 15 μL of buspirone NDS or saline into both nostrils twice a day for seven days. Two rats were untreated and served as a negative control. At the end of the experiment, animals were sacrificed, and their nasal cavities were removed and fixed in 3.8% buffered formaldehyde, pH 7.4. Sections of the nasal cavity were cut serially at 7-μm thickness and stained with hematoxylin and eosin. The sections were examined by a professional histopathologist (Authority for Animal Facilities, Hebrew University of Jerusalem, Israel) by Zeiss Axioskop 2 plus (Oberkochen, Germany). Local toxicity was assessed by evaluating the histopathological alterations in different regions of the nasal cavity (cartilage and turbinate bone, lamina propria and submucosa, mucosal epithelium and lumen).

### 2.7. Statistical Analysis

Data is reported as mean ± SD and analyzed by one-way ANOVA with the Tukey–Kramer multiple comparisons post-test or by unpaired two-tailed *t*-test. *p* < 0.05 is considered significant in all cases.

## 3. Results

### 3.1. Buspirone NDS Characterization

**Figure 1:**
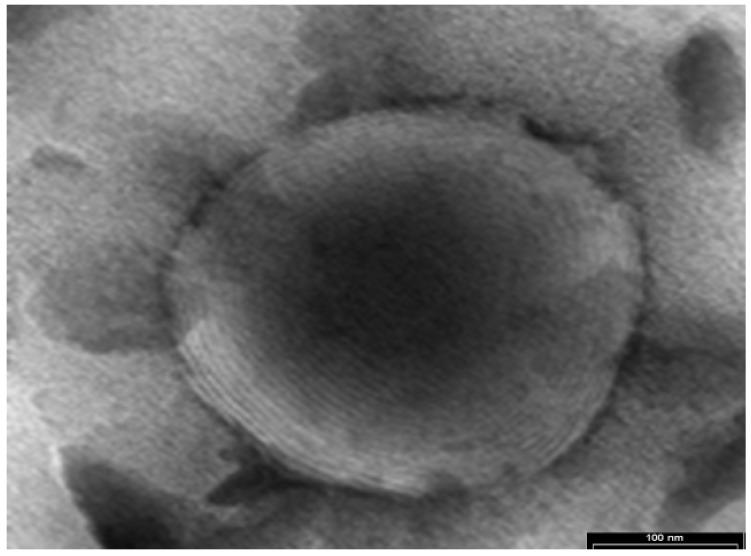
A multilamellar vesicle apparent in the transition electron (TE) micrograph of a buspirone nanovesicular delivery system (NDS) (110 k, Philips TEM CM 120 electron microscope).

**Figure 2:**
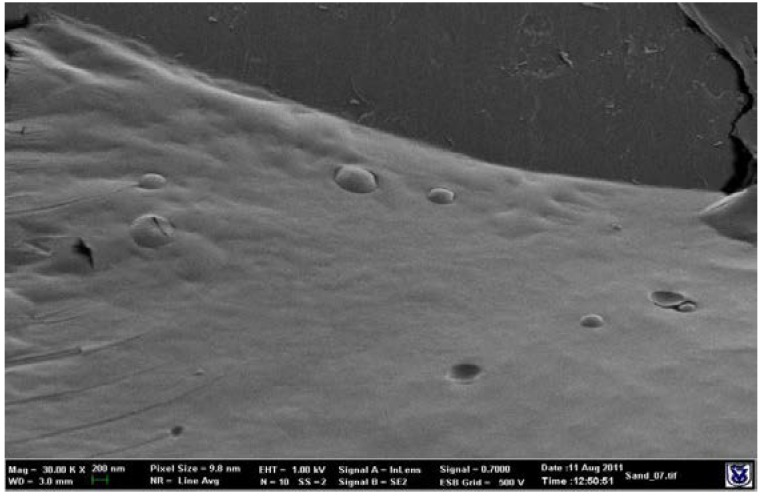
Cryo-scanning electron (cryo-SE) micrograph of a buspirone NDS (Zeiss Ultra Plus HR-SEM) showing multiple nanovesicles.

The TE and cryo-SE micrographs presented in Figure 1 and Figure 2 indicate the presence of nanovesicles in the tested samples.

The micrograph in Figure 1 shows a spherical multilamellar nanovesicle.

The mean size distribution of the vesicles obtained by DLS measurements was 370.0 ± 68.8 nm. 

Other important system characteristics were viscosity 72.7 ± 8.1 cP and pH 5.8 ± 0.2.

**Figure 3:**
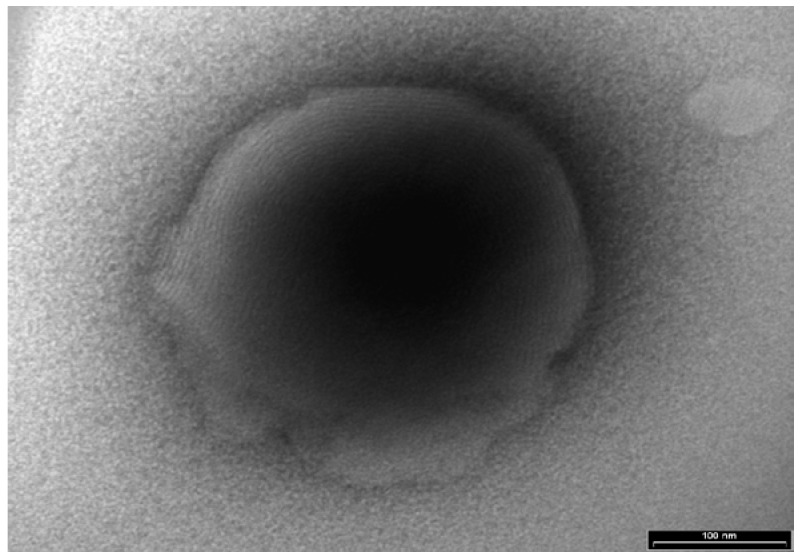
TE micrographs of buspirone NDS after three months of storage at room temperature (RT).

Stability tests results for samples kept three months at room temperature are given in Table 1 and Figure 3.

As shown in Table 1, the percentage of change in drug content, viscosity, and pH after three months’ storage at RT were less than 10% compared to the values at the initial time (zero time) storage. It is noteworthy that although the mean size distribution of vesicles decreased by 16%, the values remained in the initial nanosized range. No changes in the appearance of the vesicles were observed; the vesicles kept their spherical shape and multilamellar arrangement (Figure 3). These results suggest that the nanovesicular buspirone NDS preserved its characteristics and structure during the tested storage period.

### 3.2. Effect of Buspirone Nasal Administration on TST Values in Animal Model

The effect of buspirone NDS on TST values was tested in OVX animals.

**Figure 4:**
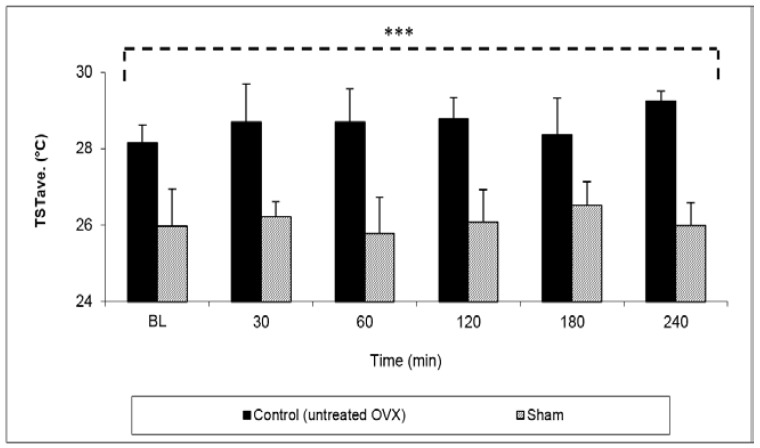
Tail skin temperature (TST) average values in untreated ovariectomized (OVX) Sprague–Dawley female rats and an untreated sham group. Data represent the mean ± SD, *n* = 8 for each group. *** *p* < 0.001 extremely significant by unpaired two-tailed *t*-test.

The first step was to validate the OVX animal model by comparing the TST values in untreated OVX rats versus intact rats (sham-operated). The TSTave (°C) values in OVX rats at all of the tested time points were significantly higher than the values obtained in sham rats with an overall mean TST of 28.8 ± 0.3 °C vs. 26.1 ± 0.3 °C, respectively (*p* < 0.001) (Figure 4).

**Figure 5:**
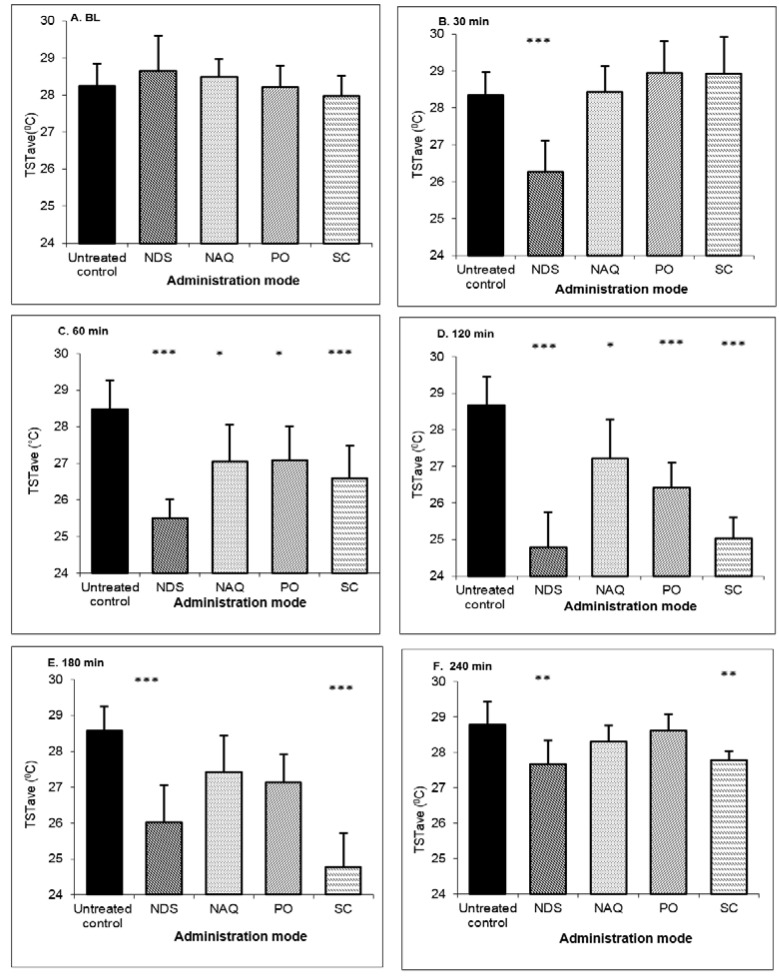
TST average values after buspirone NDS administration to OVX rats at dose 3 mg/kg compared to nasal aqueous solution (NAQ), oral administration (PO) and subcutaneous injection (SC) and in untreated control at: (**A**) baseline (BL); (**B**) 30 min; (**C**) 60 min; (**D**) 120 min; (**E**) 180 min; and (**F**) 240 min time points. Data represent the mean ± SD, *n* = 8 for each group. * *p* < 0.05, significant, ** *p* < 0.01 very significant, *** *p* < 0.001 extremely significant; compared to control (untreated OVX) by one-way ANOVA, with the Tukey–Kramer multiple comparisons post-test.

Buspirone was administrated at a dose of 3 m/kg as follows: in NDS, nasal aqueous solution, subcutaneous injection, and oral solution, and compared with results in untreated OVX rats. The TST baseline values were ≥28.5 °C in all of the OVX animal groups. The results in Figure 5 show that the nasal drug administration of buspirone NDS leads to a rapid and significant reduction in TST 30 min after treatment, achieving TSTave and ∆TST values of 26.26 ± 0.86 and −2.40 ± 0.48 °C, respectively. The treatment resulted in a statistically significant decrease in TST over the four hours of tested time as compared with PO, NAQ, or untreated OVX animals.

TST values reduction at 30 min was seen only in the treatment with buspirone NDS. At this time point, the TST values for all of the other controls were comparable to those of untreated OVX animals. At 60 min, low changes in TST were measured for the NAQ and PO groups. Further, the first significant reduction for the SC-treated group was at 60 min, indicating a relatively slow onset of action (Figure 5 and Table 2).

The calculated pharmacodynamic parameters of the above described experiments are presented in Table 2. The duration of a statistical significant effect on TST was 210 min for buspirone NDS administration and only 180 min and 60 min for SC and NAQ or PO, respectively. In addition, the absolute values of mean and maximum ∆TST were significantly higher in OVX rats treated with buspirone NDS than in the three control groups.

Further, it was interesting to compare the effect of one buspirone NDS treatment with one week of repeated subcutaneous EE.

The mean ∆TST values obtained were −2.61 ± 1.07 and −2.71 ± 0.47 for the nasal administration and the end of one week EE administration, respectively (Table 2). The TST values were near the baseline in the normal rat model.

Another important parameter is the onset of action of buspirone NDS. The evaluation was carried out by measuring the TST values of OVX rats each minute for the first 30 min following the treatment. The baseline TST value was 28.3 ± 0.5 °C; a slight increase was observed in the first five minutes following treatment, which could be a result of stress from anesthesia and administration procedures. At 10 min, a temperature reduction was measured followed by significant decrease at 15 min (*p* < 0.05) lasting to the end of experiment.

### 3.3. Buspirone Levels in Plasma and in Brain

Plasma and brain drug concentration as a function of time were measured following a single dose of 3 mg/kg buspirone nasal administration to normal rats from NDS and compared with a PO administration of a similar dose.

**Figure 6:**
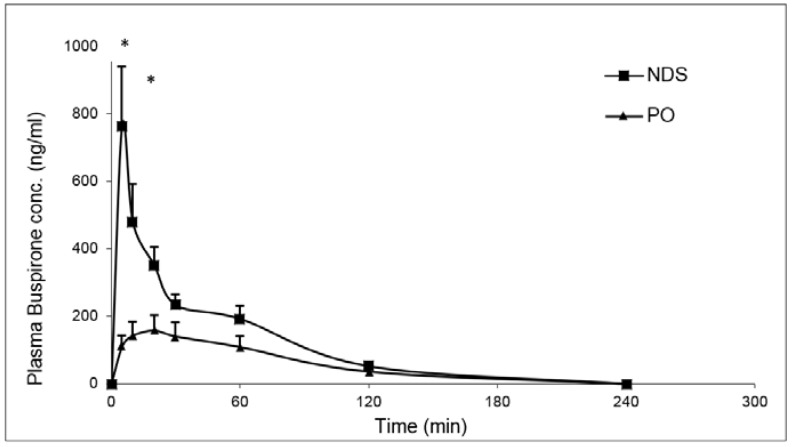
Plasma buspirone concentration-time profile after the administration of buspirone NDS to rat at a dose of 3 mg/kg compared with oral administration (PO). Data given as mean ± SD, *n* = 5 for each group. * *p* < 0.05 significant by an unpaired two-tailed *t*-test.

The results show that nasal drug administration produced a rapid increase in the drug plasma levels reaching concentrations of 764.2 ± 420.0 ng/mL and 478.2 ± 253.8 ng/mL at 5 min and 10 min post-administration, respectively. Then, the drug concentration decreased gradually 240 min after administration. Following oral administration, a relatively slow and mild increase in buspirone concentration was measured, 109.7 ± 74.9 ng/mL, 141.7 ± 95.3 ng/mL and 157.1 ± 102.9 ng/mL at 5 min, 10 min, and 20 min, respectively. A slow decrease occurred after 20 min, and the drug levels reached zero 240 min post-administration. The calculated parameters indicated that the drug nasal administration allowed for a four times higher C_max_ value than oral administration (764.2 ± 420.0 ng/mL and 181.5 ± 106.5 ng/mL, respectively) with a three times shorter T_max_ value (5.0 ± 0.0 min and 15.0 ± 7.1 min, respectively). The calculated AUC0−240 _[NDS]_ and AUC0−240 _[PO]_ values were 27515.3 ± 9104.4 and 12089.3 ± 8826.3, respectively, indicating a relative plasma bioavailability of 212.7% (Figure 6, Table 3).

**Figure 7:**
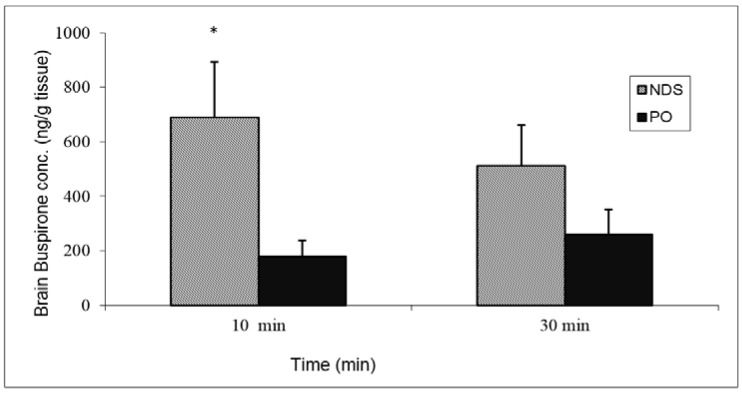
Brain buspirone concentrations after administration of buspirone NDS to rat at a dose of 3 mg/kg compared with oral administration (PO). Data represent the mean ± SD, *n* = 5 for each group. * *p* < 0.05 significant by unpaired two-tail *t*-test.

Drug quantities measured in the brain tissue at 10 min and 30 min post-buspirone NDS administration were 688.4 ± 204.7 ng/g and 511.1 ± 149.4 ng/g, respectively. It is noteworthy that the drug concentrations that were detected in the brain tissue following PO administration were lower by five and two times (179.6 ± 58.8 ng/g and 258.9 ± 91.7 ng/g at 10 min and 30 min, respectively) (Figure 7).

### 3.4. Local Safety

Histopathological analysis of the cavity and nasal tissue following sub-chronic buspirone NDS administration was assessed by comparing the nasal cavities treated with the new system or with saline and untreated rats.

No pathological findings were observed in the histopathological analysis of the nasal cavities excised from rats in the buspirone NDS and saline groups (images not shown). The results show intact mucosal epithelium, empty lumen, and no infiltration of inflammatory cells. Overall, there was no evidence of inflammation. Turbinate bone integrity was preserved. Epithelium was normal with no evidence of erosion or ulceration, and ciliated epithelium was intact. These findings are sustained by previous results obtained with tramadol NDS [7].

## 4. Discussion

Nasal administration is a nice alternative to improve the bioavailability of drugs that are poorly absorbed by the oral route. This mode of administration is generally associated with good patient compliance [4]. However, the nasal administration of some drugs may also result in low absorption due to their insufficient permeation across the nasal mucosa [18]. In previous publications, we proposed a new effective and safe nasal nanovesicular carrier for various drugs and treatments. The enhanced effect of drugs in pain and Multiple Sclerosis animal models was achieved following nasal administration using this delivery system [6,7,8].

In this work, we designed and characterized buspirone NDS, which is the nanovesicular carrier containing the drug. The effect of this system on hot flushes was evaluated in OVX rats. The OVX rat model used in the present study for pharmacodynamic evaluation is an acceptable model for estrogen deficiency-associated thermoregulatory dysfunction [2,14]. Subsequently, the elevation in TST is considered similar to menopausal hot flushes in women [19,20].

The ability of the new nasal nanovesicular system to enhance the systemic and brain delivery of buspirone was also investigated.

We found that the system is composed of spherical nanosized multilamellar vesicles, as evidenced by electron microscopy and DLS measurements. The pH of buspirone NDS was shown to be within the suitable range for nasal administration. Moreover, the system was stable for three months of storage at RT.

Treating OVX rats nasally with buspirone NDS lead to a significant reduction in TST. The effect was higher and over a longer time period than oral ornasal aqueous solutions, or subcutaneous injection.

The improved systemic and brain delivery of buspirone were proven by higher drug levels achieved in plasma and the brain (C_max_), in addition to shorter T_max_ values following administration in the nanovesicular system compared with oral administration. The efficient delivery of buspirone to the brain via the nanovesicular NDS points toward the ability of the system to target the drug to the animal brain.

It is notable that other non-hormonal drugs including serotonin/norepinephrine reuptake inhibitors, gabapentinoids, and clonidine have been considered for the management of vasomotor symptoms (VMS) to overcome the side effects of HT [21]. These drugs are usually administered via the oral route.

The treatment we suggest here presents a new approach to be further investigated for hot flushes management in cases where women experience symptoms hourly, or suffer from night sweats and sleep disturbances. It is also suggested to be helpful in menopause women suffering from VMS associated with anxiety, owing to the approved anxiolytic effect of buspirone [22]. In addition, the nasal administration of buspirone could avoid drugs interaction in the gastrointestinal tract when the oral administration of other drugs is required.

## 5. Conclusions

Buspirone that was incorporated in the new nanovesicular carrier delivered nasally to the OXV animal model for hot flushes was more efficient than the administration of the same drug dose in nasal or oral aqueous solutions and subcutaneous injection. Buspirone levels in the brain and plasma of rats following nasal administration of the drug in the new carrier were superior to those measured in oral administration. Sub-chronic nasal administration of the buspirone nanosystem has shown no pathological changes in the mucosa for the tested period.

The feasibility data generated in this investigation point toward the possibility of considering buspirone NDS for further studies, and the development of a non-hormonal treatment of hot flushes.

## 6. Patents

Touitou, E.; Godin, B.; Duchi, S. Compositions for nasal delivery. 2014. US patent 8,911,751 B2.

## Figures and Tables

**Table 1:** Stability parameters for buspirone nasal nanovesicular delivery system (NDS) at zero time and after three months of storage at RT (mean ± SD).

Time	0 Time	Three Months	% of Initial after Three Months *
Drug Content, % *w*/*w*	3.13 ± 0.05	2.88 ± 0.02	92.0
Vesicles Mean Size Distribution, nm	395.1 ± 162.9	332.6 ± 111.1	84.0
Viscosity, cP	87.0 ± 3.1	91.0 ± 4.3	104.6
pH	5.73 ± 0.10	5.85 ± 0.05	102.1

* Calculated by the following equation: (value after three months/value at 0 time) × 100%.

**Table 2:** Pharmacodynamic parameters of buspirone NDS administration to OVX rats as compared with controls (*n* = 8 for each group), mean ± SD.

System	Mean ∆TST, °C	Duration *, (min)	Max ∆TST, °C (Time, min)
NDS **	−2.61 ± 1.07	210	−3.88 ± 0.95 (120)
NAQ **	−0.46 ± 0.75	60	−1.16 ± 0.88 (60)
PO **	−0.39 ± 1.05	60	−0.58 ± 0.91 (120)
SC **	−1.86 ± 1.78	180	−3.71 ± 1.15 (180)
EE ***	−2.71 ± 0.47	240	−3.55 ± 0.20 (30)

TST, tail skin temperature; NDS, nasal nanovesicular delivery system; PO, oral administration; NAQ, nasal aqueous solution; SC, subcutaneous injection; EE, subcutaneous ethinylestradiol injection. * Duration of effect is the time at which the average TST is statistically different from the TST at baseline by one-way ANOVA, with the Tukey–Kramer multiple comparisons post-test. ** Buspirone administrated from systems at a single dose of 3 mg/kg. *** Subcutaneous ethinylestradiol administrated at a dose 0.3 mg/kg once daily for 7 days.

**Table 3:** Pharmacokinetic parameters following the administration of 3 mg/kg buspirone in NDS and PO to rat at a similar dose (*n* = 5, for each group), mean ± SD.

Pharmacokinetic Parameter	Administration Mode	
	NDS	PO
T_max_ (min)	5.0 ± 0.0	15.0 ± 7.1
C_max_ (ng/mL)	764.2 ± 420.0	181.5 ± 106.5
AUC0−240 (ng × min/mL)	25,715.3 ± 9104.4	12,089.3 ± 8826.3

NDS: nasal nanovesicular delivery system; PO: oral administration. C_max_: maximum plasma concentrations; T_max_: time at which C_max_ is achieved; AUC0−240: area under the curve from zero time to 240 min.
